# Clinical features, diagnosis and management of amoxicillin-induced Kounis syndrome

**DOI:** 10.3389/fphar.2022.998239

**Published:** 2022-10-31

**Authors:** Chunjiang Wang, Yulu Zhou, Weijin Fang, Zuojun Li, Shaoli Zhao

**Affiliations:** ^1^ Department of Pharmacy, The Third Xiangya Hospital, Central South University, Changsha, Hunan, China; ^2^ Department of Pharmacy, Hunan Provincial Maternal and Child Health Care Hospital, Changsha, Hunan, China; ^3^ Department of Endocrinology, The Third Xiangya Hospital, Central South University, Changsha, Hunan, China

**Keywords:** Kounis syndrome, amoxicilline, coronary spasm, acute coronary syndrome, hypersensitivity

## Abstract

**Background:** The available evidence suggests that amoxicillin is often associated with the occurrence of Kounis syndrome (KS). The purpose of this study is to explore the clinical characteristics of KS induced by amoxicillin.

**Methods:** We searched for case reports of amoxicillin-induced KS through Chinese and English databases from 1972 to May 2022.

**Results:** A total of 33 patients with KS were included, including 16 patients (48.5%) receiving amoxicillin treatment and 17 patients (51.5%) receiving amoxicillin-clavulanate. The median age was 58 years (range 13–82), 75.8% were from Europe and 81.8% were male. Nearly 70% of KS patients develop symptoms within 30 min after administration. Chest pain (63.6%) and allergic reaction (75.8%) were the most common clinical manifestations. Diagnostic evaluation revealed elevated troponin (72.7%), ST-segment elevation (81.2%) and coronary artery stenosis with thrombosis (53.6%). Thirty-two (97.0%) patients recovered completely after discontinuation of amoxicillin and treatments such as steroids and antihistamines.

**Conclusion:** KS is a rare adverse reaction of amoxicillin. Amoxicillin-induced KS should be considered when chest pain accompanied by allergic symptoms, electrocardiogram changes and or elevated levels of myocardial injury markers. Therapeutic management of KS requires simultaneous treatment of cardiac and allergic symptoms. Epinephrine should be used with caution in patients with suspected KS.

## Introduction

Kounis syndrome (KS) is an acute coronary syndrome caused by an allergic reaction, and first reported by Kounis and Zavras in 1991 (ounis and Zavras, 1991). Patients with a history of allergies, hypertension, smoking, diabetes, and hyperlipidemia are more likely to be affected. KS can occur at any age, but the most commonly affected age group is 40–70 years (68%) of male patients (74.3%) (Abdelghany et al., 2017). KS seems to have a geographical distribution and is mostly reported in southern Europe, especially in Greece and Turkey (Kounis, 2016). Three variants of KS have been defined. The Type I variant (coronary spasm) includes patients with normal or near-normal coronary arteries but without predisposing factors for coronary artery disease. Allergic reactions result in coronary spasm, with or without elevation of markers of myocardial injury. The Type II variant include patients with pre-existing atherosclerotic disease, acute allergy causing plaque erosion or rupture, presenting as acute myocardial infarction. The type III variant variant refers to allergic manifestations and stent thrombosis after coronary drug stent implantation (Kounis, 2016).

A variety of reasons have been found to induce KS, including many drugs, diseases, food, environmental exposure or certain other conditions (Kounis, 2016). Among them, antibiotics are the most common cause of KS, accounting for about 27%, mosquito bites account for about 23% (Abdelghany et al., 2017). Amoxicillin is a commonly used beta-lactam antibiotic and is usually associated with possible adverse events such as gastrointestinal, allergic reactions and haematological reactions (Salvo et al., 2007). KS is a rare and serious complication after the administration of amoxicillin. Knowledge of amoxicillin-induced KS is largely based on case reports. The clinical features of KS induced by amoxicillin are still unclear. The purpose of this study is to explore the clinical characteristics of KS induced by amoxicillin, and to provide a basis for the rational use of amoxicillin.

## Methods

### Search strategy

We searched the literature related to amoxicillin-induced KS by searching Chinese databases (Wanfang Data, China National Knowledge Infrastructure (CNKI), Chinese VIP) and English databases (PubMed/Medline, Embase, Web of Knowledge, OVID, Elsevier, Springer Link and Cochrane Library databases) from 1972 to May 2022. The search combined subject and free words such as amoxicillin, amoxicillin-clavulanate, Kounis syndrome, antibiotics, allergic reactions, β-lactams, thrombosis, myocardial infarction, acute coronary syndrome, coronary spasm, hypersensitivity.

### Inclusion and exclusion criteria

Inclusion criteria: case report and case analysis of KS induced by amoxicillin. The clinical data is relatively complete, including the amoxicillin application, clinical manifestations, laboratory examinations, treatment and prognosis etc. Exclusion criteria: duplicate literature, reviews, mechanistic studies, animal studies and articles for which the full text was not available.

## Data extraction

Two researchers independently conducted a preliminary screening of the literature according to the inclusion and exclusion criteria, and then the group discussed the included literature. We extract the following information of patients: region, gender, age, medical history, drug combination, amoxicillin application, indication, symptom onset time, clinical manifestations, laboratory examinations, imaging examinations, treatment and prognosis by using self-designed data extraction table.

### Statistical analysis

Statistical analyses were performed using SPSS 22.0 (SPSS Inc., Chicago, IL). Continuous data is represented by median value with ranges, counting data is represented by number of cases and percentage (%).

## Results

### Basic information

**FIGURE 1:**
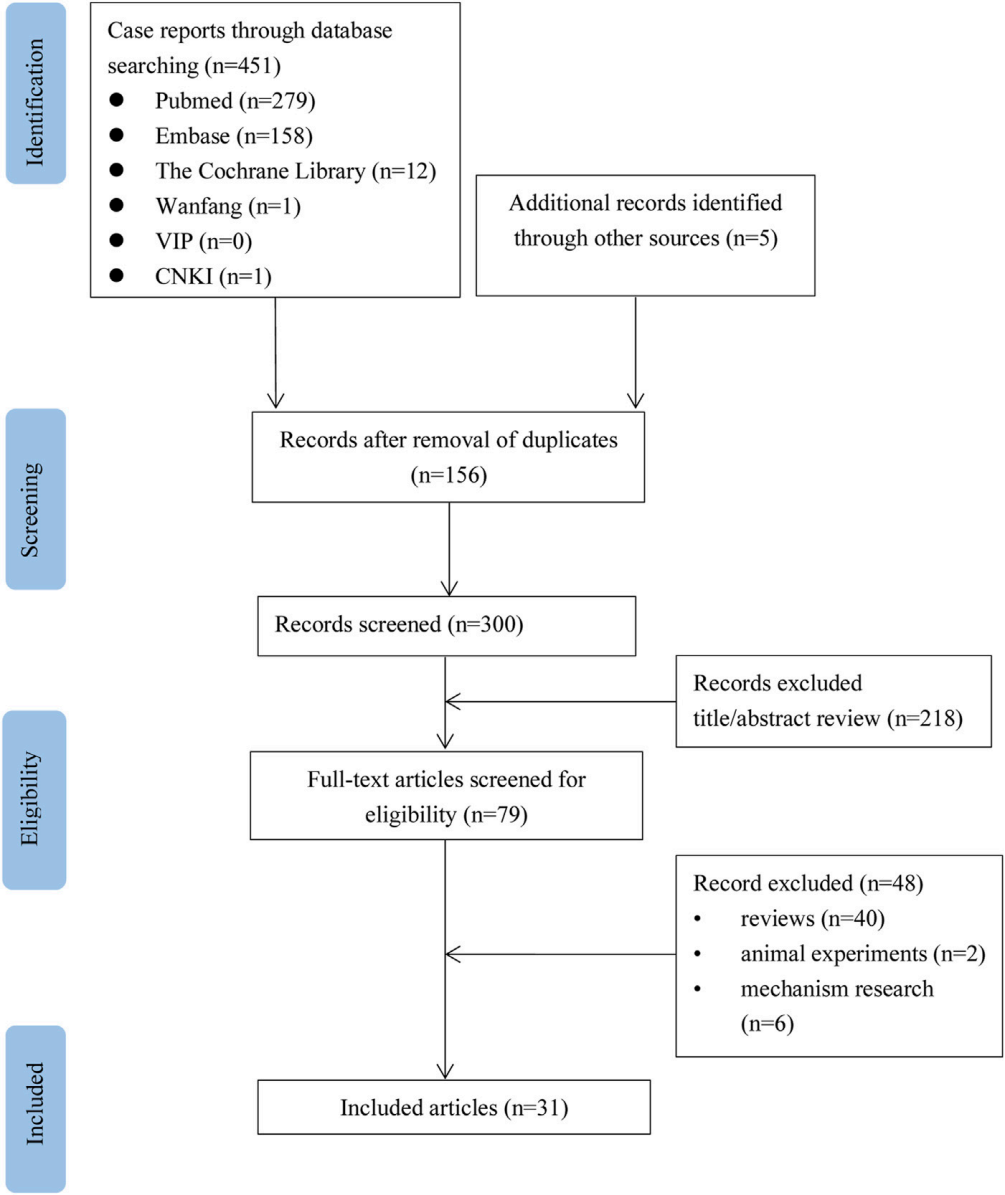
Flow chart of the study selection process for reported cases of amoxicillin-induced Kounis syndrome.

As shown in Figure 1, a total of 456 relevant studies were initially identified. One hundred and fifty-six duplicate studies were excluded. After an initial screening of titles and abstracts, 218 articles were removed. Of the remaining 79 studies, 48 were excluded from the full-text review. A total of 31 studies were included (Alemparte Pardavila et al., 1999; López-Abad et al., 2004; Moreno-Ancillo et al., 2004; Gikas et al., 2005; Del Furia et al., 2007; Tigen et al., 2007; Tavil et al., 2008; Vivas et al., 2008; Biteker et al., 2009; Caglar et al., 2011; Venturini et al., 2011; Viana-Tejedor et al., 2011; Mazarakis et al., 2012; Bezgin et al., 2013; Calf et al., 2013; Ilhan et al., 2013; Kilickesmez et al., 2013; Lombardi et al., 2013; González-de-Olano et al., 2014; Ralapanawa and Kularatne, 2015; Molina Anguita et al., 2016; Salouage et al., 2016; Shimi et al., 2016; Antonelli et al., 2017; Canpolat et al., 2017; Omri et al., 2017; Pradhan et al., 2018; Lopes and Agarwal, 2019; Moloney et al., 2019; Caragnano et al., 2020; Duarte et al., 2020). The basic information of these 33 patients is summarized in Table 1. These patients included 27 males (81.8%) and 6 females with a median age of 58years (range 13–82). These patients include 25 cases (75.8%) in Europe, 3 patients (9.1%) in Africa, 2 patients (6.1%) in Asia, and 2 patients (6.1%)) in the United States, 1 patient (3.0%) in Oceania. Amoxicillin and amoxicillin-clavulanate are primarily used for infection and dental care prophylaxis. There were 16 patients with other diseases, including hypertension in 8 patients (24.2%), dyslipidemia in 6 patients (18.2%), diabetes in 4 patients (12.1%), ischemic heart disease in 3 patients (9.1%). Eight patients (24.2%) had a history of smoking habits (Vivas et al., 2008; Venturini et al., 2011; Mazarakis et al., 2012; Lombardi et al., 2013; Salouage et al., 2016; Pradhan et al., 2018; Caragnano et al., 2020). Four patients (12.1%) had previous history of penicillin-allergy (Antonelli et al., 2017; Omri et al., 2017; Caragnano et al., 2020; Duarte et al., 2020). Five patients (15.2%) were treated with other drugs concurrently.

**TABLE 1:** Summary of clinical information of 33 patients with amoxicillin-induced Kounis syndrome.

Reference	Age/Gender	Drug	Daily dosage (g)	Route of administration	Time of symptom onset	Symptoms	Troponin I (ng/ml)	ECG	Echocardiography	Coronarography	KS type	Treatment
5	M/62	AC	0.5	oral	15 min	Unconsciousness, skin vasodilation	NA	ST elevation	NA	Stenosis	II	dopamine
6	M/56	AC	0.5	oral	30 min	CP, D, R	1.37	normal	normal	NA	NA	steroids, antihistamines, NG
7	M/32	AC	0.5	oral	2 h 15 min	dizziness, blurred vision, D, abdominal pain, anaphylaxis	45.5	ST elevation	EF 60%	normal	I	steroids, E, aspirin, UF, pethidine, NG, reteplase
8	F/70	AC	0.5	oral	Few min	P, warmth, flushing, lips and hands swelling, D, unconscious	0.31	ST elevation	hypokinesis	stenosis	II	steroids, antihistamines, aspirin, nitrates
9	M/69	AC	first dose	IV	Few min	CP, epigastric discomfort	normal	ST elevation	normal	normal	I	steroids, NG, CCB
10	F/40	AC	first dose	oral	20 min	CP, swollen extremities, R, tongue swelling, dysphagia	11.65	ST elevation	EF 50%, hypokinesis	Stenosis	II	steroids, antihistamines, tirofiban, clopidogrel, metoprolol, ramipril, atorvastatin.
11	M/64	AC	NA	oral	immediately	CP, erythema, N, V	4.48^a^	ST elevation	normal	thrombosis	II	NG, antihistamines
12	M/48	AC	2	oral	3d	CP, R, S	3.7	ST elevation	EF 40%, hypokinesis	stenosis	III	steroids, antihistamines, simvastatin, aspirin, atenolol, nitrates, clopidogrel
13	M/64	AC	1	oral	5 min	chest discomfort, unconsciousness, R, S	2.1	ST elevation	NA	stenosis	II	steroids, antihistamines, aspirin, clopidogrel, UF
14	M/71	AC	a tablet	oral	within min	V, U, P, dizziness, hypotension	0.266	ST depression	NA	NA	NA	steroids, antihistamines, E
15	F/61	AC	0.25	oral	10min	V, despair, diarrhea, dizziness, fainting	0.245	negative T-wave	normal	NA	I	steroids, antihistamines
16	M/53	AC	NA	oral	Few min	CP, U, P, ventricular, fibrillation	1,272	ST elevation	NA	NA	NA	steroids, antihistamines
17	M/60	AC	1	oral	immediately	CP, altered mental status, dizziness, P, warmth, flushing, D, U	0.0047	ST elevation	EF 30%, hypokinesis	Stenosis, occlusion	II	steroids, antihistamines, UF, clopidogrel, streptokinase
18	M/62	AC	0.5	oral	immediately	general asthenia, face erythema	0.064	ST elevation	EF 55%	stenosis	II	adrenaline, mechanical ventilation
19	M/22	AC	single	oral	1 h	CP, chest tightness	0.550	ST elevation	normal	normal	I	aspirin, UF
20	M/64	AC	1	oral	After 5 tablets	CP, weakness, S	0.14	repolarization abnormalities	NA	subocclusion	I	isosorbide dinitrate, aspirin, LMWH
20	M/57	ACA	0.875	oral	15 min	CP, N, R, P	3.78	ST elevation	EF 55%, hypokinesis	normal	I	steroids, antihistamines
20	M/58	ACA	0.875	oral	Few min	CP, R, D	normal	ST elevation	NA	normal	I	steroids, antihistamines
21	F/58	ACA	NA	oral	1 h	CP, flushing, P, warmth, facial oedema, U, dizziness	0.51	Pardee waves	NA	thrombosis	III	steroids, morphine, E, UF
22	F/56	ACA	2	oral	Immediately	chest discomfort, N, V, S, erythema, U	7.9	ST depression	EF 60%	normal	I	steroids, antihistamines, ephedrine, aspirin, clopidogrel
23	M/54	ACA	1	oral	30 min	CP, P, N	NA	ST elevation	EF 30%, hypokinesis	vasospasm	I	aspirin, clopidogrel, UF, nitrate
24	M/53	ACA	NA	oral	NA	CP, D, S, N, dizziness, cyanosed lips, U	NA	ST-elevation	NA	stenosis	II	steroids, adrenaline, aspirin, fentanyl, UF, ticagrelor
25	F/73	ACA	NA	IV	1 min	R, altered state of consciousness	2046	ST elevation	NA	stenosis	II	steroids, antihistamines, advanced life support, mechanical ventilation
26	M/74	ACA	1.2	IV	20 min	anaphylactic shock, R, P, palpitations, chest tightness, S	2.2	ST elevation	hypokinesis	NA	NA	steroids, antihistamines, adrenalin, aspirin, clopidogrel, atovastatin
27	M/43	ACA	NA	oral	NA	CP, P, erythema	NA	ST elevation	NA	normal	I	steroids, antihistamines, oxygen
28	M/61	ACA	1	oral	10 min	CP, R	0.288^a^	ST elevation	NA	normal	II	antihistamine
29	M/13	ACA	0.5	oral	30 min	CP, R	13	ST elevation	hypokinesis	normal	I	steroids, antihistamines
30	M/31	ACA	NA	oral	1 h	angina pectoris	↑	ST elevation	normal	stenosis	NA	thrombolytic therapy (tPA)
31	M/29	ACA	1	oral	NA	CP, D	29	ST elevation	normal	normal	I	steroids, antihistamines, morphine
32	M/16	ACA	1	oral	NA	ischemic pain	NA	ST elevation	normal	normal	I	NA
33	M/82	ACA	NA	IV	Immediately	abdominal pain, D, erythematous, R	NA	ST elevation	NA	normal	I	steroids, antihistamines, aspirin, UF
34	M/58	ACA	0.875	oral	30 min	CP, D, weakness, S, hypotension	normal	ST elevation	NA	normal	I	E
35	M/25	ACA	1	oral	20 min	CP, R	2.40^a^	ST elevation	hypokinesis	normal	I	NG, anti-ischemic and anti-platelet drugs

Abbreviation: AC, amoxicillin; ACA, amoxicillin and clavulanic acid; IV, intravenous; CP, chest pain; V, vomiting, N, nausea; P, pruritus; S, sweating; D, dyspnea; R, rash, U, urticaria; Electrocardiogram; LMWH, low molecular weight heparin; UF, unfractionated heparin; tPA, tissue plasminogen activator; CCB, calcium channel blockers; NG, nitroglycerine; ECG, E, epinephrine. Na, Not applicable.

^a^ Represents the value of Troponin T.

### Administration of amoxicillin

In these patients, 16 patients (48.5%) received amoxicillin and 17 patients (51.5%) received amoxicillin-clavulanate (Table 2). The daily dose of amoxicillin ranged from 0.25 g to 2 g, and the dose range of amoxicillin-clavulanate ranged from 0.5 g to 2 g. The route of administration was oral in 29 patients (87.9%) and intravenous in 4 patients (12.9%). KS had a wide range of onset times, from immediately after taking the medicines to 3 days. Symptoms occurred immediately after taking the medicines in 5 patients (15.2%), within half an hour in 18 patients (54.5%), 1 h in 3 patients (3.0%), 2 h 15 min in 1 patient (3.0%), after taking the 5th tablet in 1 patient (3.0%), and 3 days in 1 patient (3.0%). Two patients (6.1%) developed similar symptoms after taking amoxicillin in their previous medical history (Del Furia et al., 2007; Tavil et al., 2008). One patient (3.0%) took amoxycillin in the past without any related symptoms (Gikas et al., 2005). Symptoms reappeared in 1 patient (3.0%) who received amoxicillin again (Moreno-Ancillo et al., 2004).

**TABLE 2:** Basic information of 33 patients with amoxicillin-induced Kounis syndrome.

Parameter	Clinical features	Value
Sex	males	27 (81.8%)
	females	6 (18.2%)
Age	years	58 (13-82)^b^
Race	Europe	25 (75.8%)
	Africa	3 (9.1%)
	Asia	2 (6.1%)
	USA	2 (6.1%)
	Oceania	1 (3.0%)
Drug	amoxicillin	16 (48.5%)
	amoxicillin-clavulanate	17 (51.5%)
Route of administration	oral	29 (87.9%)
	intravenous injection	4 (12.9%)
Symptom onset time (29)^a^	immediately	5 (15.2%)
	within 30 min	18 (54.5%)
	1 h	3 (9.1%)
	2 h 15 min	1 (3.0%)
	3 days	1 (3.0%)
	5th tablet	1 (3.0%)
Indication	upper respiratory tract infection	9 (27.3%)
	dental infection	5 (15.2%)
	dental care prophylaxis	3 (9.1%)
	pulmonary infection	2 (6.1%)
	flu	2 (6.1%)
	urinary tract infection	2 (6.1%)
	trauma	1 (3.0%)
	systemic inflammatory response syndrome	1 (3.0%)
	na	8 (24.2%)
Medical history (16)^a^	hypertension	8 (24.2%)
	dyslipidemia	6 (18.2%)
	diabetes	4 (12.1%)
	ischemic heart disease	3 (9.1%)
	thyroid disease	2 (6.1%)
	cerebrovascular disease	2 (6.1%
	asthma	2 (6.1%)
	vesical neoplasy	1 (3.0%)
	volvulus	1 (3.0%)
	epilepsy	1 (3.0%)
Risk factors	penicillin-allergy	4 (12.1%)
	non-steroidal anti-inflammatory drugs food allergy	1 (3.0%)
	smoking	1 (3.0%)
		8 (24.2%)
combination therapy	nebivolol/hydrochlorothiazide, doxazosin, insulin, clopidogrel, captopril, furosemide, metformin, aspirin, statins, tapazole	5 (15.2%)

^a^ Represents the number of patients out of 33 on which information regarding this particular parameter was provided.

^b^ Median (minimum-maximum).

Abbreviations: na, not applicable.

### Clinical manifestations

The clinical symptoms of 33 KS patients are summarized in Table 3. The main clinical manifestations of these patients included chest pain in 21 patients (63.6%), allergic reactions (rash, pruritus, erythema) in 25 patients (75.8%), neurological adverse reactions (alteration of consciousness, dizziness) in 10 patients (30.3%), and gastrointestinal adverse reactions (nausea, vomiting, abdominal pain) in 10 patients (30.3%), dyspnea in 6 patients (18.2%), swelling (lips, hands, tongue, face) in 2 patients (6.1%). Thirteen patients had hypotension at the onset of symptoms (Alemparte Pardavila et al., 1999; López-Abad et al., 2004; Gikas et al., 2005; Vivas et al., 2008; Mazarakis et al., 2012; Kilickesmez et al., 2013; Lombardi et al., 2013; González-de-Olano et al., 2014; Ralapanawa and Kularatne, 2015; Salouage et al., 2016; Shimi et al., 2016; Duarte et al., 2020). Cardiac arrest occurred in 4 patients (12.1%) (Calf et al., 2013; Canpolat et al., 2017; Caragnano et al., 2020; Duarte et al., 2020).

**TABLE 3:** Clinical symptoms, imaging and laboratory tests of 33 patients with amoxicillin-induced Kounis syndrome.

Parameter	Clinical features	Value
Symtoms	chest pain	21 (63.6%)
	allergic reactions (rash, pruritus, erythema)	25 (75.8%)
	neurological adverse reactions (alteration of consciousness, dizziness)	10 (30.3%)
	gastrointestinal adverse reactions (nausea, vomiting, abdominal pain)	10 (30.3%)
	hypotension	13 (36.3%)
	dyspnea	6 (18.2%)
	cardiac arrest	4 (12.1%)
	chest discomfort	2 (6.1%)
	swelling (lips, hands, tongue, face)	2 (6.1%)
	chest tightness	1 (3.0%)
Electrocardiogram	ST elevation	27 (81.2%)
	depression	2 (6.1%)
	pardee waves	1 (3.0%)
	left ventricular repolarization abnormalities	1 (3.0%)
	negative T-wave	1 (3.0%)
	normal	1 (3.0%)
Echocardiography (20) ^a^	normal	13 (65.0%)
	hypokinesis	9 (45.0%)
	reduced ejection fraction	3 (15.0%)
Coronary angiography (28) ^a^	normal	14 (50.0%)
	stenosis	12 (42.9%)
	thrombosis	2 (7.1%)
	left coronary artery	8 (28.6%)
	right coronary artery	4 (14.3%)
Laboratory examination (27)^a^	troponin T	3 (11.1%)
	elevated	3 (11.1%)
	troponin I	24 (88.9%)
	normal	3 (11.1%)
	elevated	21 (77.8%)
		2.2 (0.064, 2046) ^b^
	creatine kinase	15 (45.5%)
	normal	5 (15.2%)
	elevated	10 (30.3%)
	creatine kinase-myocardial band	9 (27.3%)
	elevated	9 (27.3%)
	serum tryptase	5 (15.2%)
	elevated	5 (15.2%)
	skin prick tests	10 (30.3%)
	positive	10 (30.3%)

^a^ Represents the number of patients out of 33 on which information regarding this particular parameter was provided.

^b^ Median (minimum-maximum).

### Laboratory examination

The laboratory tests of 33 KS patients are summarized in Table 2. Laboratory exams of troponin and tryptase performed after the beginning of the episode in some patients. Of the 27 recorded cases, 3 patients (11.1%) had elevated troponin T, 3 patients (11.1%) had normal troponin I, and 21 patients (77.8%) had elevated troponin I, with a median of 2.2 ng/ml (range 0.064–2046). Creatine kinase (CK) was reported in 15 patients(45.5%), with elevations in 10 patients (30.3%) (Alemparte Pardavila et al., 1999; Moreno-Ancillo et al., 2004; Gikas et al., 2005; Tigen et al., 2007; Caglar et al., 2011; Viana-Tejedor et al., 2011; Bezgin et al., 2013; Antonelli et al., 2017; Pradhan et al., 2018; Duarte et al., 2020). Nine patients (27.3%) reported elevated creatine kinase-myocardial band (CK-MB) (Moreno-Ancillo et al., 2004; Gikas et al., 2005; Tigen et al., 2007; Tavil et al., 2008; Biteker et al., 2009; Venturini et al., 2011; Bezgin et al., 2013; Kilickesmez et al., 2013; Omri et al., 2017). The serum tryptase levels were significantly elevated in 5 patients (15.2%) undergoing examination (Biteker et al., 2009; González-de-Olano et al., 2014; Molina Anguita et al., 2016; Lopes and Agarwal, 2019; Duarte et al., 2020). The results were positive in 10 patients (30.3%) undergoing skin prick tests (Alemparte Pardavila et al., 1999; López-Abad et al., 2004; Moreno-Ancillo et al., 2004; Gikas et al., 2005; Del Furia et al., 2007; Vivas et al., 2008; Bezgin et al., 2013; Kilickesmez et al., 2013; González-de-Olano et al., 2014; Molina Anguita et al., 2016).

### Imaging examination

The imaging examinations of 33 KS patients are summarized in Table 2. Electrocardiogram (ECG) examination mainly showed ST segment elevation in 27 patients (81.2%). Very few patients showed pardee waves (3.0%), depression (6.1%), left ventricular repolarization abnormalities (3.0%) and negative T-wave (3.0%) on ECG. Only 1 patient had normal ECG (3.0%). Echocardiography in 20 patients (60.6%) at the onset of KS showed normal in 13 patients (65.0%), hypokinesis in 9 patients (45.0%), and reduced ejection fraction in 3 patients (15.0%) (Venturini et al., 2011; Canpolat et al., 2017; Caragnano et al., 2020). Coronary angiography in 28 patients showed normal in 14 patients (50.0%), stenosis in 12 patients (42.9%), and thrombosis in 2 patients (7.1%) (Viana-Tejedor et al., 2011; Salouage et al., 2016). The left coronary artery (LCA) was affected in 8 patients (28.6%), (Del Furia et al., 2007; Caglar et al., 2011; Viana-Tejedor et al., 2011; Mazarakis et al., 2012; Lombardi et al., 2013; Salouage et al., 2016; Omri et al., 2017; Moloney et al., 2019), and the right coronary artery (RCA) was affected in 4 patients (14.3%) (Tigen et al., 2007; Viana-Tejedor et al., 2011; Mazarakis et al., 2012; Canpolat et al., 2017).

### Treatment and prognosis

The treatment and prognosis of the 33 KS patients are summarized in Table 4. All patients immediately withdrew amoxicillin and amoxicillin-clavulanate after the onset of symptoms (Table 2). The remaining treatment options included steroids in 22 patients (66.7%), antihistamines in 20 patients (66.0%), epinephrine in 6 patients (18.2%), nitrate in 8 patients (24.2%), anti-platelet drugs in 14 patients (42.4%), anticoagulant drugs in 10 patients (30.3%), thrombolytic therapy in 1 patient (3.0%). Revascularization was performed in 9 patients (27.3%), including percutaneous coronary intervention (PCI) in 8 patients (24.2%), coronary artery bypass surgery (CABS) in 1 patient (3.0%) (Alemparte Pardavila et al., 1999; Del Furia et al., 2007; Caglar et al., 2011; Venturini et al., 2011; Mazarakis et al., 2012; Lombardi et al., 2013; Antonelli et al., 2017; Omri et al., 2017; Moloney et al., 2019). Thirty-two patients (97.0%) recovered completely, and only one patient (3.0%) died (Omri et al., 2017).

**TABLE 4:** Treatment and prognosis of 33 patients with amoxicillin-induced Kounis syndrome.

Parameter		Value
Treatment	discounted	33 (100%)
	steroids	22 (66.7%)
	antihistamines	20 (66.0%)
	epinephrine	6 (18.2%)
	nitrate	8 (24.2%)
	anti-platelet drugs	14 (42.4%)
	anticoagulant drugs	10 (30.3%)
	thrombolytic therapy	1 (3.0%)
	cardiac resuscitation	1 (3.0%)
	percutaneous coronary intervention	8 (24.2%)
	coronary artery bypass surgery	1 (3.0%)
Outcome	recovered	32 (97.0%)
	died	1 (3.0%)
Kounis syndrome variants	I	16 (48.5%)
	II	10 (30.3%)
	III	2 (6.1%)
	na	5 (15.2%)

Abbreviations: na, not applicable.

## Types of Kounis syndrome

Sixteen patients (48.5%) belonged to type I KS variant, 10 patients (30.3%) belonged to type II KS variant, 2 patients (6.1%) belonged to type III KS variant. The KS variant could not be identified in the remaining 5 patients (15.2%).

## Discussion

KS is an allergic acute coronary syndrome that can occur at any age, but the most commonly affected age group is 40–70 years (68%) of male patients (74.3%). Patients with a history of allergies, hypertension, smoking, diabetes, and hyperlipidemia are more likely to be affected (Abdelghany et al., 2017). Among the 33 reported cases of KS induced by amoxicillin, the majority were type I variant, the patients were mainly middle-aged men from Europe. Approximately 70% of cases occur within 30 min after administration. The diagnosis of KS mainly relies on clinical symptoms and signs as well as laboratory tests, electrocardiogram, echocardiography and coronary angiography. In addition to the typical symptoms of chest pain, allergic reactions will appear, including rash, hives. Cardiac troponin I or T and myocardial enzymes (CK, CK-MB) are important markers of myocardial injury. ECG usually showed ischemia-related ST-segment changes, of which ST-segment elevation was the most common manifestation. Coronary angiography may show spasm or stenosis of coronary vessels. The study showed that LCA was the culprit in approximately one-third of patients with coronary vasospasm or stenosis.

It is currently believed that the occurrence of KS is caused by allergic reactions in people with allergies after exposure to specific antigens. The main inflammatory cells that are involved in the development of KS are mast cells that interact with macrophages and T-lymphocytes (Fassio et al., 2016). Infiltration of activated mast cells into plaque erosion or rupture areas is a common pathway between allergic and non-allergic coronary events (Kovanen et al., 1995). These activated cells release inflammatory mediators, including histamine, neutral proteases, arachidonic acid products, platelet activating factor and heparin, etc., leading to peripheral vasodilatation, hypotension, coronary spasm, and coronary atherosclerosis erosion, rupture of plaque-like plaques or thrombosis in coronary stents (Abdelghany et al., 2017).

At present, the guideline for the treatment of KS have not been established, and the treatment recommendations are mainly derived from the experience summary of case reports. The treatment of KS should consider two aspects of acute coronary syndrome (ACS) and allergic reaction. Patients with ACS should be treated according to the ACS guidelines. Anti-allergic treatment often has a better effect in patients with type I KS variant, while patients with type II variant and III KS variant need to treat acute coronary syndromes while being anti-allergic (Abdelghany et al., 2017). Corticosteroids and H1 and H2 antihistamines can all reduce or eliminate allergy symptoms. The administration of vasodilators such as calcium channel blockers and nitrates can abolish hypersensitivity induced vasospasm. Epinephrine should be used with caution in KS, because it can aggravate myocardial ischemia, prolong the QTc interval and induce coronary vasospasm and arrhythmia (Fassio et al., 2016). Stabilizing mast cells and preventing the release of inflammatory mediators may be a new therapeutic strategy for KS. Drugs and natural molecules that stabilize mast cells include mediator antagonists, mediator biosynthesis inhibitors, leukotriene antagonists, mediator receptor blockers such as sodium nedocromil, sodium cromoglycate, ketotifen, lodoxamide, humanized IgG1 monoclonal antibodies and others which interfere with mast cell stabilization (Cevik et al., 2010).

KS has a good prognosis and can fully recover with appropriate treatment in most patients. Our research showed that amoxicillin-induced KS may have serious complications, such as cardiac arrest in 12.1% of patients and death in 3% of patients.

## Conclusion

In conclusion, KS is a rare adverse reaction of amoxicillin. Amoxicillin-induced KS should be considered when chest pain accompanied by allergic symptoms, ECG changes and or elevated levels of myocardial injury markers.

## Data Availability

The original contributions presented in the study are included in the article/Supplementary Material, further inquiries can be directed to the corresponding author.
